# SGID: a comprehensive and interactive database of the silkworm

**DOI:** 10.1093/database/baz134

**Published:** 2019-12-14

**Authors:** Zhenglin Zhu, Zhufen Guan, Gexin Liu, Yawang Wang, Ze Zhang

**Affiliations:** 1 School of Life Sciences, Chongqing University, No.55 Daxuecheng South Rd., Shapingba, Chongqing, 401331, China; 2 Khoury College of Computer Sciences, Northeastern University, 401 Terry Ave N, Seattle, WA, 98109, USA

## Abstract

Although the domestic silkworm (*Bombyx mori*) is an important model and economic animal, there is a lack of comprehensive database for this organism. Here, we developed the silkworm genome informatics database (SGID). It aims to bring together all silkworm-related biological data and provide an interactive platform for gene inquiry and analysis. The function annotation in SGID is thorough and covers 98% of the silkworm genes. The annotation details include function description, Gene Ontology, Kyoto Encyclopedia of Genes and Genomes pathway, subcellular location, transmembrane topology, protein secondary/tertiary structure, homologous group and transcription factor. SGID provides genome-scale visualization of population genetics test results based on high-depth resequencing data of 158 silkworm samples. It also provides interactive analysis tools of transcriptomic and epigenomic data from 79 NCBI BioProjects. SGID will be extremely useful to silkworm research in the future.

## Introduction

The silkworm, *Bombyx mori*, domesticated from its wild ancestry, *B. mandarina*, nearly 5000 years ago, contributes to silk industry, pest control (1, 2) and evolutionary biology. It is a promising model organism in life sciences (3). Because of its importance, the silkworm genome was sequenced and annotated in 2004 (4, 5) and supplementarily annotated in 2012 (6). According to the records in NCBI PubMed, >9000 silkworm-related works have been published. The chromosome-level assembly of the silkworm genome is accomplished in 2017 and published in 2019 (7). Meanwhile, with the development of the sequencing technology, massive silkworm transcriptomic or epigenomic data were produced (8–12). Up to now, there are already >1000 records of silkworm DNA-seq or RNA-seq data documented in NCBI SRA database. A comprehensive archiving and synthesis of these data is important to silkworm research.

Many model organisms have their own online bioinformatics analysis platform, such as TAIR for *Arabidopsis* (13), Flybase (14) for *Drosophila* and MGI (15) for mouse. Currently, SilkBase (16) is the only workable dedicated database for silkworm. It mainly focuses on the archive of sequences and is lacking in analysis tools. Ensembl Silkworm (http://metazoa.ensembl.org/Bombyx_mori) and InsectBase (17) are absence of update and without workable whole genome browser. Until now, there is still not a comprehensive online analysis platform of the silkworm.

To assist silkworm research, we developed the silkworm genome informatics database (SGID) through collecting and cataloging comprehensive genomics, transcriptomics, proteomics and epigenomics data. On the basis of previous works (7, 16, 18), we thoroughly annotated silkworm genes in the contents of function, protein structure, homolog and transcription factor (TF). We also incorporated repeat elements, population statistic tests and epigenomic analysis results into the genome browser, which will help users to get a more comprehensive picture of genome segments. We developed interactive and click-one type analysis tools in SGID, letting users to obtain one or more genes’ overall information swiftly.

## Materials and Methods

### Data processing for basic gene annotation

We used the high-quality assembly of the silkworm genome (7) as the reference. Based on gene models (2017) in SilkBase, we re-annotated all silkworm genes by UniProt (https://www.uniprot.org). Generally speaking, we BLAST the protein sequence of each gene against the UniProt protein database and took hits with significant similarity (*E*-value < 0.05 and coverage > 0.7). In this way, we made connections of the silkworm genes and UniProt proteins and obtained UniProt annotations of the silkworm genes, including Pubmed ID, EMBL ID, Proteomes ID, Pfam (http://pfam.xfam.org), Interpro (http://www.ebi.ac.uk/interpro), Gene Ontologies (GOs), Kyoto Encyclopedia of Genes and Genomes (KEGG, https://www.genome.jp/kegg) and so on.

To validate gene expression in protein level, we manually collected all sequenced silkworm peptides referred in published proteomics related works and did alignments of them and all silkworm genes. We filtered out results with cutoffs of *E*-value < 0.05 and coverage > 0.8. If the predicted protein of one gene matches two or more peptides, we consider this gene has expression evidence.

We used CD-HIT (19) to search for homologous genes, requiring identity > 50% and coverage > 70%, and identified 1064 gene clusters. We did multiple alignments of the sequences within each cluster by MUSCLE (20), and built the phylogenetic tree by FastTree 2.1 (21) with the parameter ‘-boot 5000’ to test trees’ likelihoods. We called repeat elements by RepeatMasker (http://www.repeatmasker.org) and predicted TFs by the pipeline referred in AnimalTFDB 3.0 (22).

### Subcellular localization and structure prediction

Annotations from UniProt only cover part of the silkworm proteins. Thus, we re-do protein function and structure prediction for all genes. We predicted subcellular locations of ASFV proteins through CELLO v2.5 (23) and transmembrane helixes within proteins’ sequences using TMHMM 2.0 (24). The output images were converted into PNG format for display in websites by Magick (www.imagemagick.org).

We BLAST all protein sequences against the PDB database (http://www.rcsb.org) and extracted significant alignment results as we did for UniProt. We also did protein structure prediction by InterproScan (25) and put significant results into SGID. We used SignalP (26) to predict signal peptides for silkworm proteins.

### Gene Ontology

We put the GO from InterproScan, UniProt and SilkBase together as SGID GO data set. We made alignments of the silkworm genes and KEGG silkworm proteins and extracted the hits with *E*-value < 0.05 and identity > 0.9. In this way, we made connections between the silkworm genes and KEGG proteins. We also extracted KEGG pathway information from KEGG and made connections between pathways and silkworm genes using KEGG protein ID as a bridge. We obtain Entrez IDs of silkworm genes by KOBAS (27).

To enable a gene search using old gene models (4, 28), we made connections between old gene models and SilkBase gene models 2017. Like we did for KEGG proteins, we made alignments of predicted proteins between old gene models and gene models 2017 and selected the best hit of each alignment.

### Pre-processing of transcriptomic and epigenomic data

We collected transcriptomic and epigenomic data of silkworm-related projects from NCBI. For transcriptomes, we classified them into three categories, ‘DEG’ (differentially expressed genes), ‘Stage’ and ‘Tissue’. ‘DEG’ means the project is to identify differentially expressed genes in different experimental conditions. ‘Stage’ means the project is to observe gene expression at stages of different time points. Tissue means the project is to obtain expression profiling in different tissues. Following the standard RNA-seq analysis protocol (29), we mapped transcriptomic reads onto the reference genome by bowtie (30) and called Fragments per Kilobase Million (FKPM) by cuffnorm (31).

For epigenomic data, according to experimental methodologies, we classified them into ChIP-Seq, Bisulfite-Seq and miRNA. ChIP-Seq stands for combining chromatin immunoprecipitation (ChIP) assays with next-generation sequencing. Bisulfite-Seq is the use of bisulfite treatment of DNA before routine sequencing to determine the pattern of methylation (32). Small RNA means small RNA sequencing. For ChIP-Seq data, we used Bowtie to align reads onto the reference genome and inspected signatures by MAC2 (33). We used Bismark (34) to pre-process Bisulfite-Seq data. We aligned small RNA reads onto the reference genome by Bowtie. Epigenomic analysis results are converted into bigWig format by the UCSC Genome tool bedGraphToBigWig for display in terminals.

### Identification of domestication genes

We used Bowtie to map the genome resequencing data of 142 domesticated and 16 wild silkworm samples, including PRJDB4743, PRJNA402108 and an unpublished resequencing data (depth = ×30) of 15 silkworm samples produced by our lab, onto the reference genome and made bam files by SAMtools (35). To illustrate the evolution pressure at a genome-wide scale, we slid along the silkworm genome with a window size of 2000 bp and a step size of 200 bp. In each sliding window, we calculated Pi, Theta (36), Tajima’s D (37) and the composite likelihood ratio (CLR) (38) by ANGSD (39) and SweapFineder2 (40). We also did the four population genetics test for each gene and made coalescent simulation (41) ranking test (CSRT) (42) according to silkworm domestication mode (43). We called domestication genes in a strict method, requiring a Tajima’s D_domesticated_ < −1, a CSRT < 0.05, a Tajima’s D_domesticated_ < Tajima’s D_wild_, a Tajima’s D_min, domesticated_ > top 5% point value in ascending order, a Fst_max_ > the top 5% point value in descending order and a CLR_max, domesticated_ > the top 5% threshold in the whole genome. ‘Min’ or ‘max’ indicates the minimal or max value in the genic region extended by 10% of gene length to accommodate the situation that the 5′ or 3′ terminals of a gene is under evolutionary forces. A subscript of ‘domesticated’ and ‘wild’ means the population genetics test is performed on domesticated or wild silkworms. We also appended identified domestication genes in (44) and (8) to our domestication genes dataset. We searched for genes possibly under balancing selection in the criteria that a Tajima’s D _domesticated_ > the top 5% point value in ascending ranking, a Tajima’s D_wild_ < 0.5, a Tajima’s D_domesticated_ > 1 and a CSRT < 0.95.

### Genome browser and analysis tools

The genome browser of SGID is developed based on an open source population genetics visualization and analysis package SWAV (swav.popgenetics.org). We used MSAViewer (45) to show multiple alignments of homologous proteins, and phylotree (46) to display phylogenetic trees of gene clusters. The fuzzy text search in the home page is compatible with gene ID, gene name and gene function annotations. The alignment search tools in SGID are Perl codes to parse BLAT (47) or NCBI BLAST results (48). The interface to exhibit gene expression is built upon D3 and JQuery. The overall web structure is Mysql + PHP + CodeIgniter (www.codeigniter.com) + JQuery (jquery.com).

## Result and Discussions

### The biological data in SGID

Out of the 16 880 gene models predicted in the high-quality assembly of the silkworm genome (7), 13 551 are of function annotations in SilkBase, leaving 3329 with unknown function. To make annotations of genes more comprehensive, SGID incorporated protein information from UniProt and successfully annotated the functions of 15 594 genes, within which 2962 are of function descriptions for the first time. For a lot of genes, SGID gives not only simple descriptions, but also information on function details, chemical properties, related publications, protein structure, topologies, pathways and GOs. In addition to the available GO annotations of 9147 genes in SilkBase, SGID newly labeled GO IDs for 5521 genes. Besides, SGID made KEGG annotations for 16 028 genes and Entrez IDs for 16 320 genes. These are important for research, especially for gene set function enrichment analysis (Table 1).

**Table 1:** A summary of the data in SGID

Item	Description	Cov.	Previous
Genome	High-quality assembly of the silkworm genome in chromosome level (7)		The same as Kawamoto et al. and Mita et al. (7, 16)
Gene models	16 880 in total, with 12 752 correlated with old gene models (4, 28)		The same as Kawamoto et al. and Mita et al. (7, 16)
Gene function annotation	15 594 genes are of function annotations	92.4%	80.3% in Kawamoto et al. and Mita et al. (7, 16)
	4937 gene feature annotations	22.0%	NA
	201 309 annotations from InterproScan	93.9%	NA
	8730 distinctive GO lists	86.9%	54.2% in Kawamoto et al. and Mita et al. (7, 16)
	16 028 correlated KEGG Gene IDs	96.4%	NA
	138 KEGG pathways	16.5%	NA
	16 320 correlated Entrez IDs	96.7%	NA
Biophysics and chemistry	2487 EC numbers	13.8%	NA
	329 biophysicochemical properties	0.6%	NA
	2445 catalytic activity annotations	12.0%	NA
	1743 cofactor information annotations	7.5%	NA
Topology	20 378 subcellular localization annotations	99.9%	NA
	2878 genes with transmembrane regions	17.0%	NA
	1960 genes with signal peptides	11.6%	NA
Proteomics and protein structure	12 394 real peptides from experiments validated 2999 protein coding genes	17.8%	NA
	9844 genes significantly correlated PDB protein structures	58.3%	NA
	1 730 892 correlated EMBL IDs	92.3%	NA
	17 762 correlated Gene3D IDs	57.2%	NA
	112 275 correlated Interpro IDs	86.9%	NA
	6257 CDD annotations	29.0%	NA
TFs	704 items	4.2%	NA
Repeat elements	571 401 segments, with 28 519 DNA transposons, 190 316 LINE, 13763 LTR and 179 435 SINE		In accordance with Osanai-Futahashi et al. (53)
Transcriptomics	306 samples from 41 projects		NA
Epigenomics	187 samples from 38 projects		NA
Populations genetics	Sliding widow analysis results based on 158 silkworm genomes		NA

‘Cov.’ denotes the coverage of genes with corresponding annotations in total genes. ‘Previous’ denotes the comparison of SGID and previous work. ‘NA’ denotes that related data is not available in previously built silkworm databases, such as SilkBase, Ensembl Silkworm and SilkDB.

**Figure 1:**
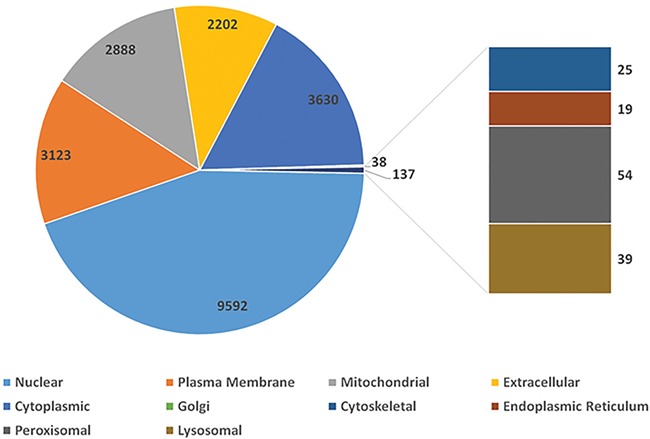
The distribution of silkworm genes in different cellular subunits.

Using peptide sequences from published experiments, we validated 2999 protein coding genes. They are of proteomics evidence. To depict one gene’s function in a cell, SGID provides information on gene’s subcellular localization and topology prediction. More than half (9592, 56.8%) of the silkworm genes are located in the nuclear (Figure 1), and 2878 genes (17.0%) have transmembrane regions. Furthermore, 1960 silkworm genes are predicted to have signal peptides. Encouragingly, 9844 silkworm proteins are of PDB matches with *E*-value < 0.05, which infers that more than half (58.3%) silkworm expressed proteins have structural information. External links to UniProt Proteomes, PRIDE (https://www.ebi.ac.uk/pride), Pfam, Interpro, SUPFAM (http://supfam.org), Gene 3D (http://gene3d.biochem.ucl.ac.uk), Protein Model Potal (49) and PANTHER (http://www.pantherdb.org) are also provided and they are helpful to understand the protein structure and related functions of one gene.

As a domesticated insect, the silkworm is important in evolution research. Domestication genes are the genes with functions to underlie one or more domestication-related traits. They usually underwent positive selection and are of complete or near-complete fixation of causative mutations in all domestic lineages (50, 51). Totally, we identified 569 domestication gene candidates (for details, see the Materials and Methods section). Users can view and inspect theses domestication genes by a SGID tool named ‘Population Genetics’. Population genetics test results are also displayed in the genome bowser, where users can do sliding window analysis of interested genomic or genic segments. We also identified 81 genes possibly under balancing selection, which are evolutionary forces that cause elevated levels of nucleotide polymorphism exceeding neutral levels, maintaining multiple alleles at higher-than-expected frequencies (52).

SGID includes transcriptomic data of 41 projects and epigenomic data of 38 projects, respectively. For transcriptomes, 28 are ‘DEG’, 9 are ‘Stage’ and 4 are ‘Tissue’ as we described in Materials and Methods. SGID includes 704 TFs belonging to 68 TF families. It also has 571 401 repeat segments covering 27.5% of the silkworm genome, which is generally in accordance with previous records (53). There are more retrotransposons (93%) than DNA transposons (7%). For retrotransposons, most are long interspersed nuclear elements (LINE) (46%) and short interspersed nuclear elements (SINE) (44%).

### The genome browser in SGID

**Figure 2:**
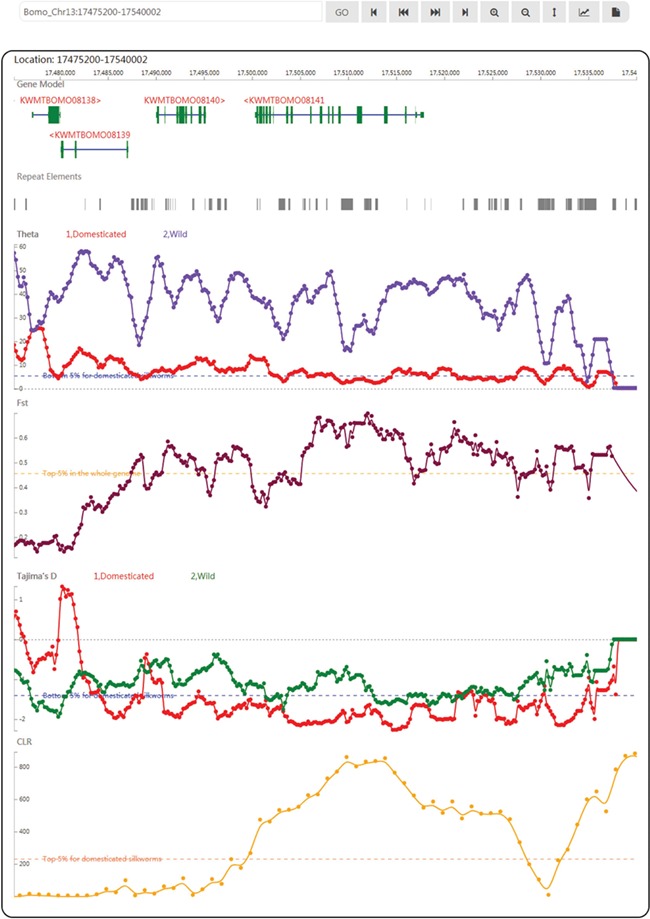
A snapshot of the genome browser of SGID with KWMTBOMO08141 in the center. Below are tracks of population genetics test results.

In SGID’s genome browser page (Figure 2), users can view the silkworm genes, repeat elements and population genetics test tracks subsequently. An input box and a list of buttons above the browser allow users to move, zoom in and zoom out, setting focus bar, generating figures or downloading the data of one track. A click onto a gene figure will take users to the gene detail page. Clicking on one point of some track will raise a dialog displaying the value at the point. Except for a genome browser, SGID also provides a browser to view epigenomic data. In the browser, users could view gene regulation signals at some specific genome position.

**Figure 3:**
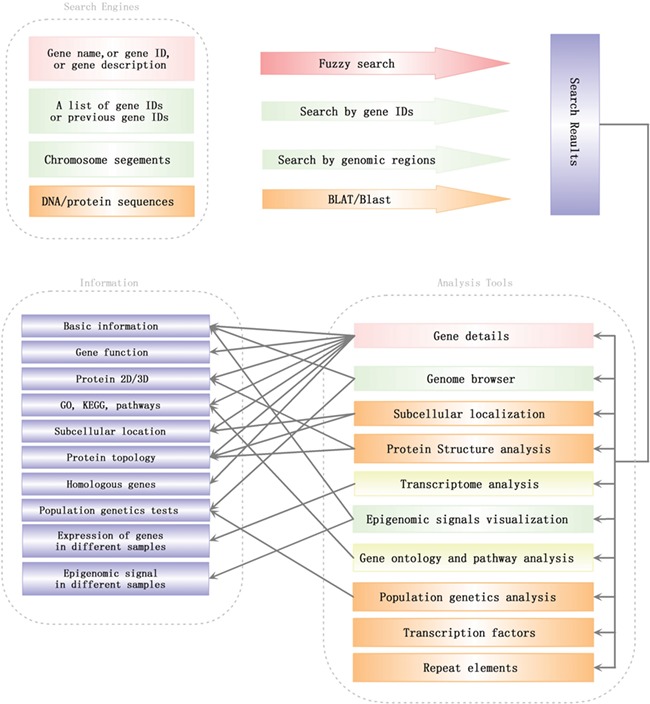
Search engines and analysis tools in SGID. Lines with arrows pointed out a general analysis flow in SGID.

### Retrieve genes’ information from SGID

As a one-click type platform, SGID offers to search genes by a gene ID of SilkBase gene models 2017 or old gene models (4, 28), a gene name, a gene function or even a brief description. In the page displaying search results, there is a list of gene information buttons within each result list. With the buttons, users can jump to view gene details, a gene in genome browser, GO and pathway, gene expression, regulation elements, gene structure and population genetics analysis results in one page or in a new window (Supplementary Figure S1A). In the detail page of each gene, aside from basic annotations (such as gene name, description, subcellular location and sequences, Supplementary Figure S1B), six information groups are listed subsequently, including ‘Summary’, ‘Ontologies’, ‘Topology’, ‘Population Genetics’, ‘Multiple Alignment’ and ‘Gene Tree’. ‘Summary’ mainly includes information resulted from protein sequence analysis (Supplementary Figure S1C). ‘Ontologies’ display a gene’s annotation on GO, KEGG Function, KEGG Pathway and PANTHER (Supplementary Figure S1C). In the part of ‘Topology’, transmembrane regions are listed and marked in a diagram (Supplementary Figure S1D). If one gene’s protein product is of signal peptide, the region of the signal peptide will also be listed and marked. ‘Population Genetics’ listed five population genetic test results (Pi, Theta, Tajima’s D, CLR and CSRT) and will give an interpretation about evolutionary forces. ‘Multiple Alignment’ and ‘Gene Tree’ displayed the multiple alignments of homologous genes at protein level and the phylogenetic tree produced based on the alignment.

To facilitate users to analyze a list of genes, SGID also offers to generate a list of gene information buttons through inputting a list of gene IDs, separated by semicolons or line feeds. With the buttons, users can jump to some information view page directly like they do in search result page as referred above. Analogously, users can input a list of chromosome positions and obtain a list of genomic information links, with which users can view the genome browser or the epigenomics browser swiftly. Users can also choose to browse in gene lists to access information or to retrieve the whole data at the download page.

### SGID analysis tools

To help users to visit data more quickly, we developed a list of analysis tools in SGID. As shown in the home page, ‘Gene Ontology’ is a tool to retrieve GO, KEGG or Entrez numbers using a list of gene IDs. ‘Transcriptome’ is a tool to view the expression of several genes in different experiment conditions, tissue or development stages. The results will be displayed in a heatmap figure. Stopping the mouse cursor at one cell of the heatmap will display the FKPM value of one gene at an experiment condition. The project’s name is listed at the top right and users can click it to view the project’s description. ‘Protein Structure’, ‘TF’, ‘Population Genetics’, ‘Repeat Elements’ and ‘Subcellular localization’ are interactive search tools, with which users can obtain a group of genes or items with some similar biological properties. ‘Cluster’ listed the 1064 gene clusters we identified. A summary of SGID search engines and analysis tools is shown in Figure 3.

### KWMTBOMO08141, a case study

KWMTBOMO08141, BGIBMGA001085 in the previous annotation (4, 5), is a domestication gene referred in (8), Bmor_03834. Through searching in the home page, we found this gene is of other 17 functional-related members as transient receptor (Supplementary Figure S1A). Using the buttons listed below, genes in the search result page, we can obtain genes’ information one by one. In the detail page, we found this gene is of a full name ‘transient receptor potential-gamma protein’ (54–56) and an alternative name ‘Transient receptor potential cation channel gamma’. The gene is annotated to be located in plasma membrane (Supplementary Figure S1B) and function in interacting preferentially with trpl and to a lower extent with trp (Supplementary Figure S1C). Encouragingly, the protein product of this gene is validated by experiment peptides (Supplementary Figure S1B) and of significant similarity to a real protein structure 5Z96 recorded in PDB (Supplementary Figure S1C). In GO, we obtained the GO and KEGG IDs of this gene and found KWMTBOMO08141 plays roles in the pathway of phototransduction in cell membrane (Supplementary Figure S1C). In topology, this gene has six transmembrane regions (Supplementary Figure S1D), which is in accordance to its subcellular localization prediction. As a domestication gene, KWMTBOMO08141 has low Tajima’s D (−1.949945) and high CLR (629.851816). In the genome browser, we observed a CLR peak at the gene’s region (Figure 2) and a higher CLR peak at the right (Supplementary Figure S1E), indicating there may be genetic hitchhiking effects in this case. In transcription analysis, we found the expression of this gene is higher in brain than other tissues (Supplementary Figure S1F) and affected by ectopic expression of ecdysone oxidase (Supplementary Figure S1G). Through scanning this gene in the SGID epigenomics browser, we observed that epigenomic signals within the genic region disappear in some cell lines (Supplementary Figure S1H).

## Conclusion

SGID is informative and user friendly. Under the idea of ‘Click-one’, SGID integrated different biological data and made them connective. SGID allows to search genes in fuzzy mode and to do analysis of more than one gene simultaneously. SGID pre-analyzed available transcriptomic data and developed a search tool to view the expression of genes in different conditions. Tools similar to SGID made the initial bioinformatics analysis of silkworm projects more efficient. With the advancement in sequencing and experiments of the silkworm, more and more data will be incorporated into SGID, making the platform to be more and more powerful.

## Data availability

All SGID data are publicly and freely accessible at http://sgid.popgenetics.net. Feedback on any aspect of the SGID database and discussions of the silkworm gene annotations are welcome by email to zhuzl@cqu.edu.cn.

## Author contributions

Z.L.Z. developed the web interface of the database. Z.Z.L., Z.G., G.L. and Y.W. collected and compiled the data and performed the analysis. Z.L.Z. and Z.Z. wrote the manuscript, conceived the idea and coordinated the project.

## Supplementary Material

Supplementary_Figures_baz134
