# Supplementary material for: SGID: a comprehensive and interactive database of the silkworm
Source: Database (Oxford). 2019 Dec 14;2019:baz134. doi: 10.1093/database/baz134 (PMC6911161; doi:10.1093/database/baz134)
Supplement: Supplementary_Figures_baz134 [file supplementary_figures_baz134.pdf]

A.

Bomyx mori | transient receptor | SEARCH

Found: 18 items

Gene	Short Name	Full Name
KWMTBOMO00115 (BGIBMGA002131)	dTRPA1	Transient receptor potential cation channel subfamily A member 1 + More
Annotation	transient_receptor_potential_cation_channel_subfamily_A_member_1_[Bombyx_mori]	
Description	Essential for thermotaxis by sensing environmental temperature. Receptor-activated non-selective cation channel involved in detection of sensations such as temperature. Involved in heat nociception by being activated by warm temperature of about 24-29 degrees Celsius.	
Gene Information · Genome Browser · Gene Ontology and Pathway · Transcriptional Analysis · View Epigenomics Data · Protein Structure · Population genetics · Close		
KWMTBOMO00118 (BGIBMGA002131)	dTRPA1	Transient receptor potential cation channel subfamily A member 1 + More
Annotation	transient_receptor_potential_cation_channel_subfamily_A_member_1_[Bombyx_mori]	
Description	Essential for thermotaxis by sensing environmental temperature. Receptor-activated non-selective cation channel involved in detection of sensations such as temperature. Involved in heat nociception by being activated by warm temperature of about 24-29 degrees Celsius.	

B.

Gene	KWMTBOMO08141 Validated by peptides from experiments
Pre Gene Modal	BGIBMGA001085
Annotation	PREDICTED:_transient_receptor_potential-gamma_protein_[Bombyx_mori]
Location	Bomo_Chr13(-):17500270-17517839 View in EpiBrowser
Full name	Transient receptor potential-gamma protein
Alternative Name	Transient receptor potential cation channel gamma
Location in the cell	PlasmaMembrane Reliability : 3.028

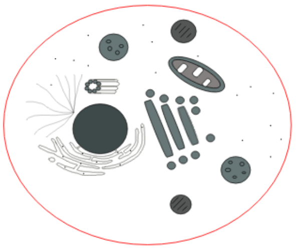

C.

Summary

Description	A light-sensitive calcium channel that is required for inositide-mediated Ca(2+) entry in the retina during phospholipase C (PLC)-mediated phototransduction (By similarity). Forms a regulated cation channel when heteromultimerized with trpl.
Subunit	Interacts preferentially with trpl and interacts to a lower extent with trp.
Similarity	Belongs to the transient receptor (TC 1.A.4) family.<br>Belongs to the transient receptor (TC 1.A.4) family. STrpC subfamily.
Keywords	ANK repeat Calcium Calcium channel Calcium transport Complete proteome Ion channel Ion transport Membrane Reference proteome Repeat Sensory transduction Transmembrane Transmembrane helix Transport Vision
Feature	chain Transient receptor potential-gamma protein
Uniprot	H9IV05 A0A212EVK1 A0A2H1WAA9 A0A2A4J9Y4 A0A2W1BXK2 A0A182RRP8 + More
Pubmed	19121390 22118469 28756777 12364791 20966253 26227816 + More
EMBL	BABH01000036 BABH01000037 BABH01000038 AGBW02012175 OWR45522.1 ODYU01007315 + More
Proteomes	UP000005204 UP000007151 UP000218220 UP000075900 UP000075920 UP000075902 + More
PRIDE	H9IV05 B7YZW4 Q9VJJ7
Pfam	PF08344 TRP_2 + More
Interpro	IPR020683 Ankyrin_rpt-contain_dom + More
SUPFAM	SSF48403 SSF48403 + More
Gene 3D	1.25.40.20 1.20.1560.10
CDD	cd00204 ANK
ProteinModelPortal	H9IV05 A0A212EVK1 A0A2H1WAA9 A0A2A4J9Y4 A0A2W1BXK2 A0A182RRP8 + More
PDB	5Z96 E-value=0, Score=1787

Ontologies

KEGG	101736324 K04967 transient receptor potential cation channel subfamily C member 4 | (RefSeq) transient recepto
PATHWAY	04745 Phototransduction - fly - Bombyx mori (domestic silkworm)

D.

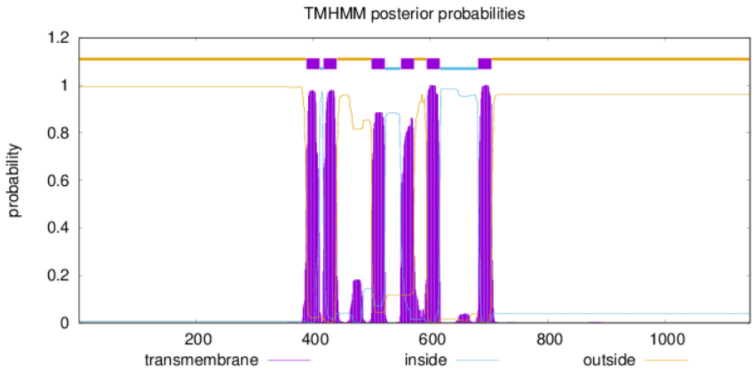
TMHMM posterior probabilities
probability
1.2
1
0.8
0.6
0.4
0.2
0
200
400
600
800
1000
transmembrane
inside
outside

E.

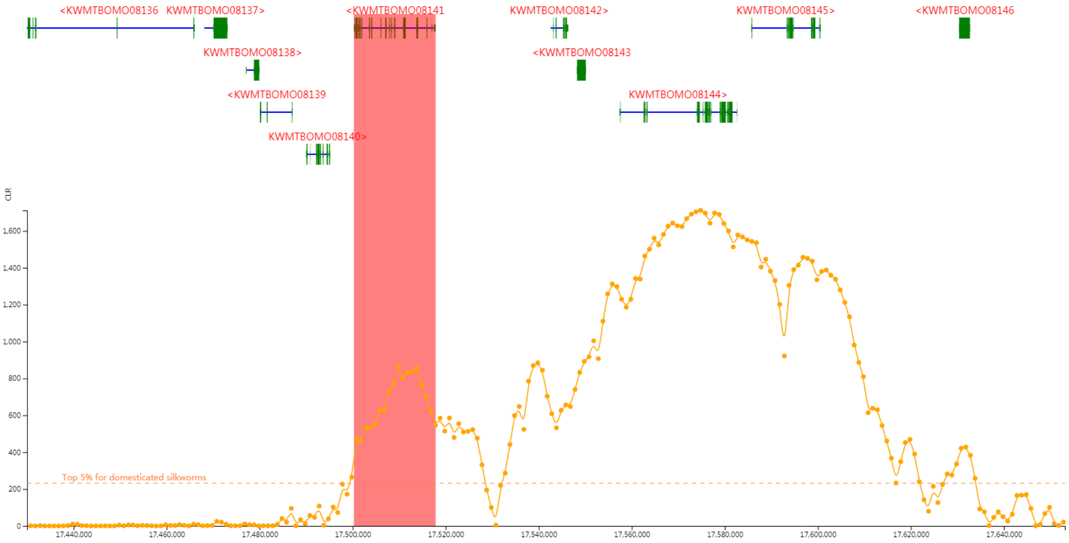
<KWMTBOMO08136
KWMTBOMO08137>
<KWMTBOMO08141
KWMTBOMO08142>
KWMTBOMO08145>
<KWMTBOMO08146
KWMTBOMO08138>
<KWMTBOMO08143
<KWMTBOMO08139
KWMTBOMO08144>
KWMTBOMO08140>
CLR
Top 5% for domesticated silkworms

F.

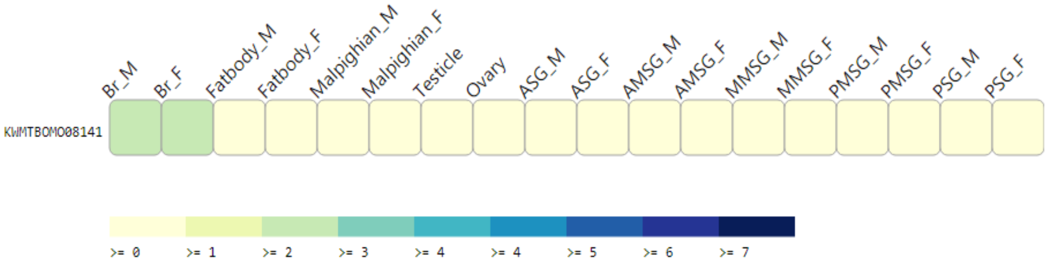
Br_M
Br_F
Fatbody_M
Fatbody_F
Malpighian_M
Malpighian_F
Testicle
Ovary
ASG_M
ASG_F
AMSG_M
AMSG_F
MMSG_M
MMSG_F
PMSG_M
PMSG_F
PSG_M
PSG_F
KWMTBOMO08141
>= 0
>= 1
>= 2
>= 3
>= 4
>= 4
>= 5
>= 6
>= 7

G.

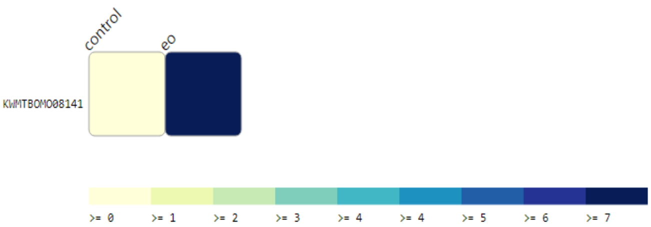
control
eo
KWMTBOMO08141
>= 0
>= 1
>= 2
>= 3
>= 4
>= 4
>= 5
>= 6
>= 7

H.

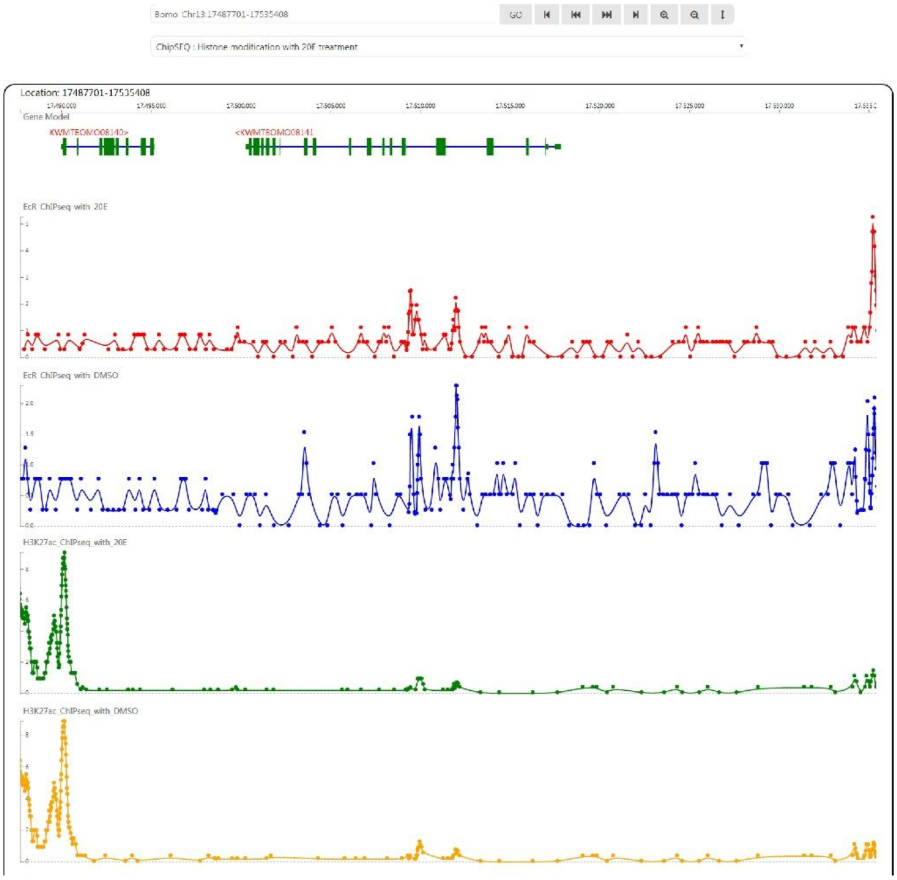

Figure S1. An example showing the online analysis of KWMTBOMO08141 in SGID. A is a snapshot of the search result for “transient receptor”. The result page lists correlated gene followed by links to gene details, genome browser, gene ontology and pathway, transcriptional analysis, epigenomic signal visualization, protein structure and population genetics analysis results. B is a snapshot of the gene detail page. Subcellular location of the gene is colored in red in a diagram following the basic information. C is the “summary” and “Ontology” parts in the detail page. D is a snapshot of the topology prediction in SGID. E is a CLR analysis figure generated by SGID genome browser with KWMTBOMO08141 marked by a focus bar colored in red. F is the expression of the gene in different tissues (PRJNA284192). M, male. F, female. Br, brain. ASG, anterior silk gland. AMSG, anterior middle silk gland. MMSG, middle middle silk gland. PMSG, post middle silk gland. PSG, post silk gland. G is the expression of the gene in the middle silk gland (PSG) of normal (control) and ecdysone oxidase overexpressed (eo) samples (PRJNA272381). H. A snapshot (partly displayed) of epigenomic signals at KWMTBOMO08141 in 4 cell lines (EcR, H3K27ac, H3K4me1 and H3K4me3) treated with DMSO or 20E (PRJNA450142).
